# PacBio high-fidelity near full-length genome sequencing for HIV-1 quasispecies: methodological framework and validation

**DOI:** 10.3389/fmicb.2025.1738027

**Published:** 2026-01-13

**Authors:** Bo Zhu, Xiaorui Wang, Hanping Li, Yongjian Liu, Lei Jia, Xiaolin Wang, Jingyun Li, Bohan Zhang, Jingwan Han, Wei Ma, Lin Li

**Affiliations:** 1 Department of Epidemiology, School of Public Health, Cheeloo College of Medicine, Shandong University, Jinan, Shandong, China; 2 State Key Laboratory of Pathogen and Biosecurity, Academy of Military Medical Sciences, Beijing, China

**Keywords:** dual infection, HIV-1, near full-length genome, PacBio Revio, quasispecies, sequencing

## Abstract

This study aimed to develop an optimized sequencing workflow for HIV-1 near full-length genome (NFLG) quasispecies, integrating the PacBio Revio platform with a streamlined bioinformatics pipeline to enable precise characterization of the genetic heterogeneity and dynamic evolution of HIV-1 quasispecies. To this end, the HIV-1 NFLG was divided into three segments for high-fidelity amplification and PacBio sequencing. This method achieved stable amplification (positivity rate >80%) in samples with a viral load >1,000 copies/mL and demonstrated applicability to China’s five major epidemic strains and unique recombinant forms (URFs). The adoption of sample-specific asymmetric barcode sequences improved cost-effectiveness, enabling efficient sequencing of 300 to 1,000 samples per sequencing cell. The entire amplification-sequencing workflow exhibited minimal systematic errors (recombination rate <3.5%, average base variation rate <0.1%). Following bioinformatic processing including filtering, clustering, and screening, the sequencing data yielded multiple accurate viral quasispecies sequences with their approximate abundance profiles. Validation studies demonstrated excellent concordance with results from conventional single-genome amplification (SGA) and Sanger sequencing, while showing superior performance in quasispecies-level dual infection detection and recombinant pattern identification. In conclusion, this study developed a PacBio HiFi-based sequencing workflow for HIV-1 NFLG quasispecies, which exhibits robust high sensitivity, accuracy, and reproducibility. This approach provides a powerful tool for deepening understanding of HIV-1 evolution, dissecting genetic diversity, tracing transmission chains, and facilitating precision antiretroviral therapy.

## Introduction

Human Immunodeficiency Virus type 1 (HIV-1) exhibits extraordinary genetic diversity driven by its high replication rate and the absence of proofreading in its reverse transcriptase (Preston et al., 1988; Roberts et al., 1988). This is a key mechanism for evading host immune responses and developing drug resistance. Furthermore, the virus establishes complex quasispecies within the host: A quasispecies consists of a cluster of closely related yet genetically distinct viral genomes that coexist within a single individual. This diversity enables the virus to evade immune clearance, acquire drug resistance, and maintain persistent infection, thereby posing significant challenges to vaccine development and antiviral therapy.

The continuous emergence of novel recombinant strains represents one of the major drivers of the expanding genetic diversity of HIV-1. Since 1990, the proportion of global HIV-1 infections accounted for by novel recombinant strains has surged dramatically, rising from 9.3% (1990–1999) to 22.8% (2010–2015). East Asia, including China, leads globally with approximately 80.5% of infections caused by these recombinants (Hemelaar et al., 2019). Over the past two decades, numerous novel recombinants have emerged, and at least 193 circulating recombinant forms (CRFs) have been identified to date (Li H. et al., 2024; Chen et al., 2025; Los Alamos National Laboratory, 2025). Studies confirm that viral quasispecies in individuals with superinfection (multiple subtypes) can continuously generate new recombinant strains, which are then transmitted to others (Redd et al., 2013; Gao et al., 2021). Accurately characterizing the genetic heterogeneity and dynamic evolution of HIV-1 populations is therefore critical for understanding pathogenesis and clinical outcomes (Castro et al., 1988; Coffin, 1995; Madlala et al., 2023). However, achieving this objective has long been hindered by the heavy reliance on short-segment sequencing techniques (e.g., drug resistance profiling) in previous studies. In contrast, near-full-length genome (NFLG) sequencing of HIV-1 quasispecies offers unique and compelling advantages in accurately and comprehensively analyzing quasispecies genetic heterogeneity, thereby facilitating the identification of novel recombinant strains and mixed infections (Salazar-Gonzalez et al., 2009; Ode et al., 2015; Li H. et al., 2024; Li Y. et al., 2024; Liu et al., 2024; Chen et al., 2025). Beyond this, it supports more precise tracing of viral strain evolution and reconstruction of transmission chains, while serving an irreplaceable function in elucidating the origin and dissemination patterns of recombinant strains, ultimately providing high-resolution data critical for molecular epidemiological studies (Banin et al., 2019; Gao et al., 2021). Furthermore, by systematically characterizing the genetic diversity and variation patterns of quasispecies, this approach can offer target information for HIV-1 vaccine design that more closely aligns with the actual evolutionary characteristics of the virus, thereby helping to address challenges such as immune escape caused by viral mutations in vaccine development. Additionally, it advances our understanding of viral pathogenetic mechanisms, such as the molecular processes underlying persistent viral infection. Consequently, NFLG sequencing of HIV-1 quasispecies is urgently needed to track viral population dynamics, identify emerging recombinant strains, optimize clinical diagnosis and treatment strategies, and support both HIV-1 vaccine development and mechanistic research into viral pathogenesis.

However, current HIV-1 sequencing technologies exhibit significant limitations. Conventional approaches such as Sanger sequencing and short-read next-generation sequencing (NGS), while useful for initial genotyping and drug resistance testing, are inadequate for deciphering the genetic architecture of quasispecies. Single-genome amplification (SGA)-based Sanger sequencing has long been the most commonly used method for HIV-1 quasispecies sequencing and reconstruction. However, it is limited by its low throughput, high cost, and labor-intensive nature, and its sensitivity for identifying low-frequency variants is inferior to that of high-throughput sequencing. Although NGS has made significant contributions to identifying the presence of quasispecies and potential mutations in specific short genomic fragments, short read lengths hinder the reconstruction of individual quasispecies viral genomes and lack the ability to capture co-occurring mutations. Reliance on assembly or alignment for genome reconstruction also introduces biases that lead to sequence distortion. Such blind spots can result in suboptimal treatment regimens, thereby accelerating the spread of drug-resistant and recombinant strains (Lazzari et al., 2025; Ma et al., 2025).

Third-generation sequencing (TGS), with its long-read capability, offers a promising technological avenue for HIV-1 quasispecies research. However, existing TGS-based approaches remain insufficient. Nanopore sequencing, plagued by high error rates complicates quasispecies identification, requiring complex error-correction workflows (Lu et al., 2016; Wright et al., 2021; Mori et al., 2022). While PacBio technology has been employed to distinguish intact viruses from defective ones and detect complex co-occurring resistance mutations, a systematic study leveraging PacBio high-fidelity (HiFi) reads to characterize HIV-1 quasispecies structure remains lacking. Furthermore, a standardized, simplified, and reproducible bioinformatics pipeline for deriving viral quasispecies sequences, their abundance data, and evaluating sequencing accuracy, has yet to be established. These limitations significantly impede the broader application of TGS in HIV-1 molecular epidemiology.

**Figure 1:**
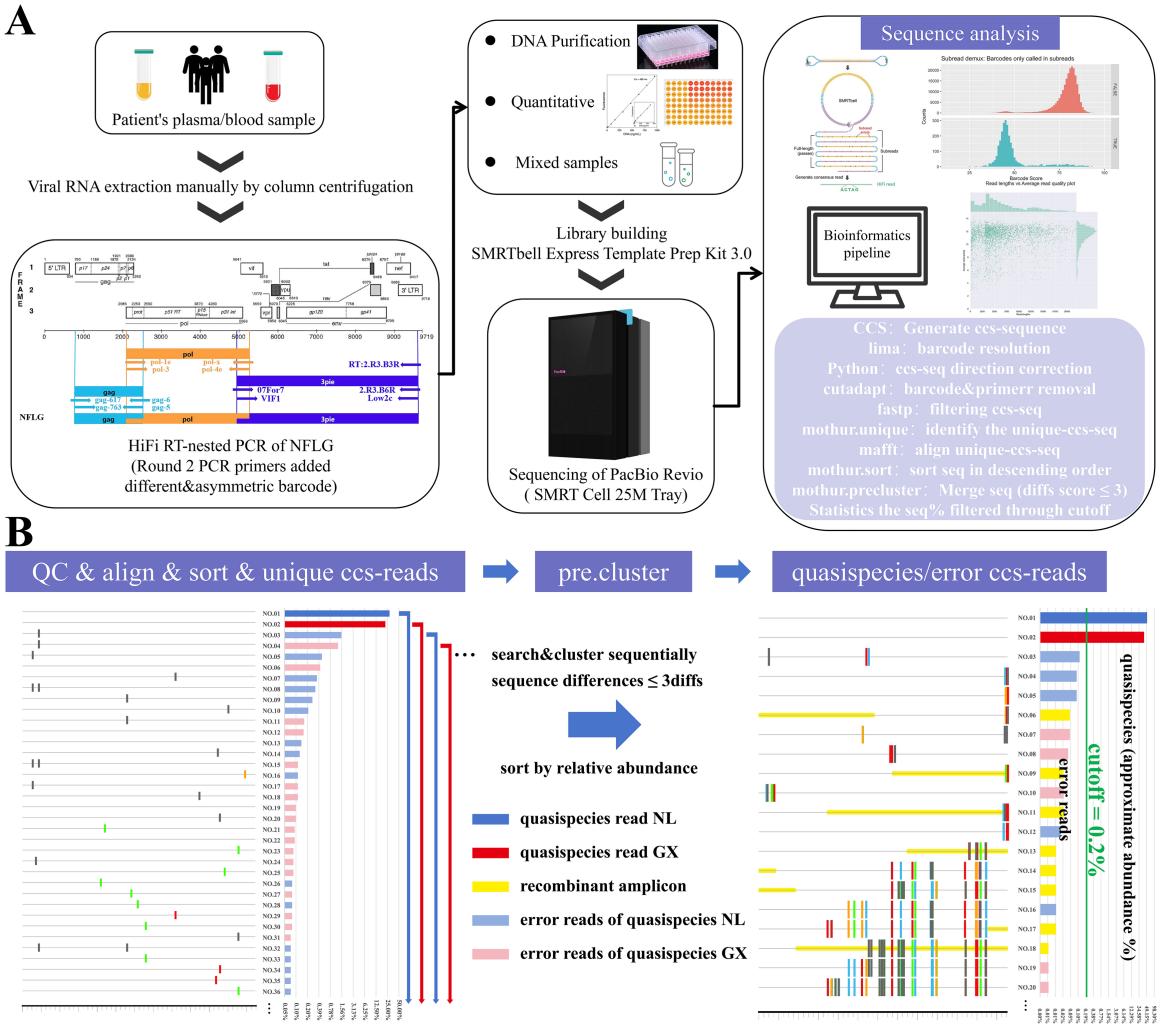
Schematic representation of HIV-1 NFLG quasispecies analysis via PacBio HiFi sequencing and bioinformatics pipeline. **(A)** Amplification, sequencing, and bioinformatics analysis pipeline. **(B)** Schematic of quasispecies extraction methods in bioinformatics pipeline (using a standard sample as an example).

This study develops an optimized HIV-1 NFLG quasispecies sequencing workflow incorporating the PacBio Revio platform and a streamlined bioinformatics pipeline (Figure 1). We refined the entire process from viral RNA extraction and amplicon design to quasispecies abundance resolution. Analysis of standard sequencing samples demonstrated low overall read variation (systematic error), with most errors easily corrected by the bioinformatics pipeline of this method. Compared to traditional Sanger sequencing, PacBio sequencing showed excellent consistency and significant superiority. Additionally, this method demonstrated excellent efficacy in acquiring quasispecies in large-scale studies. Application of this protocol to 894 clinical samples obtained 28,994 viral quasispecies sequences and abundance data of 728 samples. It identified 164 novel recombinant strains and 50 dual infections previously undetected by conventional methods, revealing complex patterns of intrahost viral population heterogeneity. Our findings demonstrate that this platform offers exceptional sensitivity and accuracy, establishing it as a critical new tool for elucidating HIV-1 evolution, dissecting genetic diversity, tracing transmission chains, and facilitating molecular diagnostics to guide precision antiretroviral therapy.

## Materials and methods

### Research objects and collection of samples

Research subjects (plasma sample) were derived from three sources: (a) Individuals newly diagnosed with HIV-1 in Shenzhen from 2011 to 2016, provided by the Shenzhen Center for Disease Control and Prevention. (b) Individuals newly diagnosed with HIV-1 in Hebei Province from 2019 to 2022, provided by the Fifth Hospital of Shijiazhuang. (c) Individuals newly diagnosed with HIV-1 in Beijing from 2023 to 2024, provided by the General Hospital of the People’s Liberation Army. Among the included subjects, at least 50 cases were allocated to each of the six categories, encompassing HIV-positive individuals‌ infected with China’s five major epidemic strains (CRF01_AE, CRF07_BC, CRF08_BC, CRF55_01B and B) and the URFs strains (Vrancken et al., 2020; Williams et al., 2023). Exclusion criteria were applied to individuals not belonging to the three major risk groups: men who have sex with men (MSM), heterosexuals (HES), and people who inject drugs (PWID). Infected individuals lacking gene sequence data of the HIV-1 *pol* region (HXB2: 2253–3441,1189 bp) sequence data were also excluded (Zhang et al., 2021). To evaluate the sensitivity of the amplification assay, we ensured the inclusion of at least 100 HIV-positive individuals with available viral load data. All participants signed written informed consent prior to the sample collection and completed the standardized questionnaire containing demographic data.

### High-fidelity amplification for NFLGs

Viral RNA was extracted from plasma samples using the QIAamp Viral RNA Mini Kit (Qiagen, Valencia, CA, USA, cat. no. 52904). To enhance the positive rate of subsequent amplification, we extracted duplicate samples and performed co-elution to enrich viral RNA. As shown in Figure 1A, specific primers were used for reverse transcription to generate cDNA to the NFLG of HIV-1, which was divided into three fragments: *gag* (HXB2: 617–2620, 2004 bp), *pol* (HXB2: 2029–5240, 3212 bp), and the 3′ half-genome (including *vif*, *vpr*, *tat*, *rev*, *vpu*, *env*, *nef*, and the partial 3′ LTR region; hereafter referred to as 3pie) (HXB2: 4875–9610, 4736 bp). Among them, the first two shorter fragments were reverse transcribed using the PrimeScript™ IV 1st strand cDNA Synthesis Mix (TaKaRa, cat. No. 6215B), while the longer 3pie fragment was processed with the Invitrogen SuperScript™ IV First-Strand Synthesis System (cat. No. 18091050). All three fragments were amplified by high-fidelity (HiFi) nested PCR. PrimeSTAR® Max DNA Polymerase Ver.2 (TaKaRa, cat. No. R047A) was used for the first round of amplification, and PrimeSTAR® GXL Premix Fast Dye plus (TaKaRa, cat. No. R052B) was employed for the second round. Specific primers are listed in Supplementary Table S1, and thermocycling parameters are provided in Supplementary material. In the second round of amplification, the forward and reverse primers each containing 16-base barcodes were used, with distinct combination patterns detailed in Supplementary Figure S1. Amplified DNA products were purified using the TIANquick N96 Purification Kit (cat. no. A4992872). Following quantification with the Quant-iT™ PicoGreen™ dsDNA Assay Kit (cat. no. P11496), products from all samples corresponding to the same fragment were pooled in equal molar amounts (Figure 1A).

### Sensitivity evaluation of NFLG HiFi PCR: viral load limit detection via serial dilutions of HIV-1 NL4-3

To standardize the evaluation of the amplification assay’s sensitivity and the lower limit of viral load detectable by our segmented amplification method, we used the NL4-3 viral supernatant as the experimental material. After quantifying its viral load, we prepared six logarithmic dilutions (10–10^6^ copies/ml) to generate standardized viral samples (8 replicates per dilution). Perform NFLG amplifications for each sample and calculate the amplification positivity rate (Li et al., 2022).

### Library preparation and sequencing‌

Pooled amplicons from the three genomic fragments underwent library preparation using SMRTbell® prep kit 3.0 (PacBio, 102-141-700), followed by sequencing on the PacBio Revio platform at Novogene (Tianjin, China). More specifically, amplicons of the 3pie fragment amplicons were sequenced on ‌2 SMRT Cell‌s, while those of the *gag* and *pol* fragments were sequenced on ‌3 and 4 SMRT Cells‌, respectively.

### Bioinformatics processing pipeline

‌Figure 1A’s right panel displays a schematic diagram of the Bioinformatics Processing Pipeline. Original polymerase reads‌ were trimmed at adapter regions and filtered to remove adapter sequences, yielding ‌subreads‌. These subreads were filtered using a standard criterion: ‌minimum length of 50 bp‌. Subsequently, HiFi reads‌ are generated from the filtered subreads using the ‌CCS (Circular Consensus Sequencing) software‌. We employed the following quality thresholds: ‌min-passes = 3‌ (a minimum of 3 cycles of the sequencing reaction in a single ZMW well to ensure read consistency) and ‌min-rq = 0.99 (indicating that the read quality score is ≥ 0.99, corresponding to a base error rate of ≤ 0.1%)‌, ensuring ‌all reads achieve Phred quality score of Q20 or higher (Phred quality score ≥20, corresponding to a base-calling error probability of ≤1%) ‌.

After sequencing, raw data were merged and demultiplexed using ‌*lima* v2.13.0‌. To minimize sample misclassification during barcode demultiplexing, ‌stringent quality filtering criteria‌ with minimum thresholds were implemented. All CCS-reads underwent ‌orientation normalization ‌using Python and ‌primer sequence trimming‌ with ‌cutadapt‌ v3.5 (Martin, 2011). Quality control was performed using ‌fastp v0.20.1 (Chen et al., 2018), which ‌discarded CCS reads‌ where ‌ > 1% of bases had a Phred quality score (Q) < 40‌. Unique sequences were generated using the ‌mothur.unique module‌, followed by ‌multiple sequence alignment‌ with ‌MAFFT v7.490‌ (Katoh and Standley, 2013; Schloss, 2020). These sequences were ‌sorted in descending order of abundance‌ using the ‌mothur.sort module‌ and ‌preclustered‌ (‌via the mothur.precluster module‌) with a threshold of ‌ ≤ 3 nucleotide differences (diffs)‌. Unique sequences ‌exceeding the abundance cutoff value (0.2%) abundance‌ in the preclustered dataset were extracted to form the ‌final quasispecies sequence dataset [Supplementary materials (markdown_file)]. The threshold for diffs and the cutoff value were determined through sensitivity analysis of quasispecies extraction from the standard sample.

### Identification and evaluation of amplicon recombination and variation in amplification-sequencing workflow

To assess sequence variations induced by systemic errors throughout the entire amplification-sequencing workflow, we generated reference standards by mixing two plasmids (pGX002 and pNL4-3) at predefined ratios (pNL:pGX = 7:3; 8:2; 9:1; 19:1; 29:1; 49:1; 99:1; 199:1)—a design intended to simulate quasispecies with distinct abundance levels. Subsequently, these eight groups of standard samples underwent ‌triplicate HiFi amplification‌ for all three fragments and were co-sequenced with all other experimental samples.

To comprehensively assess recombination-induced variations across the entire amplification-sequencing workflow, encompassing both (i) inter-amplicon recombination events occurring during PCR amplification, and (ii) sequencing read recombination resulting from co-occupancy of zero-mode waveguide (ZMW) by multiple amplicons (particularly P2 reads undetectable by pore occupancy monitoring), we conducted systematic recombination analysis using sequencing data of the three genomic fragment (*gag*, *pol* and 3pie) derived from the eight standard samples. Given the pronounced genomic divergence between pNL4-3 and pGX002 (exhibiting >10 nucleotide variations across any 400 bp segment; Supplementary Figure S2), we implemented an initial preclustering step for filtered unique reads, using a threshold of 10 nucleotide differences. This strategy effectively enhanced computational efficiency while reliably retaining recombinant reads. Subsequent recombination analysis and statistical assessments were performed on all consolidated read data.

### Maximum likelihood phylogenetic reconstruction

First, multiple sequence alignment was conducted with MAFFT. Next, the optimal model for phylogenetic inference was identified as GTR + I + G incorporating 4 substitution rate categories via the SMS algorithm. Subsequently, maximum-likelihood phylogenetic trees were inferred using PhyML 3.0 (Guindon et al., 2010). Finally, phylogenetic tree visualization and refinement were performed with FigTree v1.4.4 and iTOL v6.4.

### HIV-1 subtype identification and recombination analysis

The sequences of the three fragments were initially uploaded to the web-based quality control (QC) tool from the Los Alamos HIV Database and COMET HIV-1^1^ for preliminary subtyping, with the QC tool providing RIP and HIV BLAST results for comprehensive analysis. For sequences yielding conflicting or ambiguous identification results, they were subsequently submitted to the web-based jpHMM tool,^2^ and the breakpoint patterns were compared with those from the CRFs database in the Los Alamos HIV Database^3^ to determine final subtype; sequences exhibiting recombination patterns inconsistent with any known subtype or CRF were classified as unclassified recombinant forms (Zhang et al., 2006). For unclassified recombinant forms, further recombination analysis was performed by locally running the jpHMM tool with default parameters to batch-calculate breakpoint data, which was then converted to HXB2 positions and used to generate breakpoint plots for each sequence. While the localized jpHMM tool occasionally failed due to high indel variability in *gag*/*env* regions, these sequences were reanalyzed using RIP for breakpoint recalculation, with results confirmed by RDP4 and SimPlot v3.5.1.0; minor discrepancies were resolved by prioritizing RIP outcomes (Lole et al., 1999; Martin et al., 2015). Samples containing quasispecies with distinct subtypes/recombinant forms were classified as dual infections.

### Statistical analysis

Group-wise comparisons of proportion were analyzed using Pearson’s chi-square test of independence. Normality of differences in paired samples was assessed via the Kolmogorov–Smirnov (K-S) test. For non-normally distributed paired data, the Wilcoxon signed-rank test was applied. A *p*-value of < 0.01 was served as the threshold for statistical significance across all tests. All statistical analyses were conducted in R version 4.2.3.

## Results

### Amplification efficiency, sensitivity, and specificity

A total of 894 newly diagnosed HIV-1-infected individuals were enrolled in this study, among whom 119 cases possessed documented viral load information. Demographic and relevant clinical background information are summarized in Table 1. HiFi PCR successfully amplified 770 *gag*, 757 *pol*, and 767 3pie segments from the plasma samples, respectively. Among these, 728 samples (81.4%) showed positive amplification for all three segments, enabling NFLG recovery. The amplification positive rates of each group are shown in Table 1. Amplification failure in some samples may be attributed to: (a) undetectably low viral load; (b) extracted RNA or reverse-transcribed cDNA falling outside the quantifiable range of the assay kits; (c) suboptimal primer specificity for a minority of samples during PCR. Furthermore, nonspecific bands on agarose gel electrophoresis were observed during amplification of the *pol* and 3pie fragments in a very small subset of these samples (Supplementary materials). However, fine-tuning of template concentrations successfully generated a single specific band in all affected samples.

**Table 1:** Demographic characteristics of HIV-1 infected individuals.

Characteristic	Category	NFLG positive (total = 728), *n* (%)	NFLG negative (total = 166), *n* (%)	Positive rate
Gender	Male	635 (87.2)	142 (85.5)	82%
	Female	93 (12.8)	24 (14.5)	79%
Age (years)	<18	11 (1.5)	2 (1.2)	85%
	18–25	113 (15.5)	18 (10.8)	86%
	26–35	262 (36.0)	63 (38.0)	81%
	36–45	195 (26.8)	34 (20.5)	85%
	46–60	109 (15.0)	27 (16.3)	80%
	>60	27 (3.7)	15 (9.0)	64%
	Unknown	11 (1.5)	7 (4.2)	61%
Marital status	Single	341 (46.8)	58 (34.9)	85%
	Married/Partnered	258 (35.4)	61 (36.7)	81%
	Divorced/Separated/Widowed	71 (9.8)	17 (10.2)	81%
	Unknown	58 (8.0)	30 (18.1)	66%
Education level	College/University	157 (21.6)	21 (12.7)	88%
	High School/Vocational	168 (23.1)	38 (22.9)	82%
	Junior High School	259 (35.6)	57 (34.3)	82%
	Primary School	60 (8.2)	17 (10.2)	78%
	Illiterate/Preschool	5 (0.7)	0 (0.0)	100%
	Unknown	79 (10.9)	33 (19.9)	71%
Year of diagnosis	2011	39 (5.4)	5 (3.0)	89%
	2012	81 (11.1)	10 (6.0)	89%
	2013	22 (3.0)	4 (2.4)	85%
	2014	195 (26.8)	31 (18.7)	86%
	2015	117 (16.1)	31 (18.7)	79%
	2016	76 (10.4)	5 (3.0)	94%
	2020	47 (6.5)	19 (11.4)	71%
	2021	58 (8.0)	19 (11.4)	75%
	2023	93 (12.8)	42 (25.3)	69%
Transmission route*	HES	399 (54.8)	93 (56.0)	81%
	MSM	260 (35.7)	59 (35.5)	82%
	PWID	69 (9.5)	14 (8.4)	83%
Subtype	B	66 (9.1)	19 (11.4)	78%
	CRF01_AE	208 (28.6)	44 (26.5)	83%
	CRF07_BC	197 (27.1)	51 (30.7)	79%
	CRF08_BC	74 (10.2)	20 (12.0)	79%
	CRF55_01B	166 (22.8)	31 (18.7)	84%
	Other CRFs	2 (0.3)	0 (0.0)	100%
	URFs	15 (2.1)	1 (0.6)	94%

^*^Transmission route. HES, heterosexual contact; MSM, men who have sex with men; PWID, people who inject drugs.

Analysis of amplification rates for 119 samples with available viral load data revealed robust NFLG amplification in samples with viral loads exceeding 1,000 copies/mL. However, inconsistent amplification rates were observed in samples with viral loads ranging from 0 to 1,000 copies/mL (Supplementary Table S2). To validate these observations and further characterize the assay’s performance, we evaluated the sensitivity and specificity of the HiFi PCR assay using pNL4-3 plasmid-transfected progeny viruses with predefined viral loads (see supplementary Table S2 for full data). This experimental setup demonstrated that our assay exhibited significantly superior amplification efficiency in viral supernatant samples relative to that observed in plasma samples. It may be attributed to the greater complexity of HIV strains in plasma samples and potential viral load degradation during storage. Notably, all viral supernatant samples yielded a single specific band on agarose gel electrophoresis—confirming high amplification specificity. In contrast, a subset of plasma samples exhibited nonspecific bands in 3pie region amplification products (the agarose gel electrophoresis is additionally provided in Supplementary material), though this issue was resolved through fine-tuning of cDNA template input. However, these had a negligible impact on subsequent sequencing analysis. Nevertheless, a consistent decline in NFLG amplification positivity rates was consistently observed across all samples with viral loads below 1,000 copies/mL.

Collectively, these results indicate that this optimized amplification efficiency represents an exceptional standard in the field of the NFLG amplification research (Feng et al., 2013; Liu et al., 2024; Ma et al., 2025).

### Quality-controlled CCS reads enable high-resolution HIV-1 quasispecies analysis

**Figure 2:**
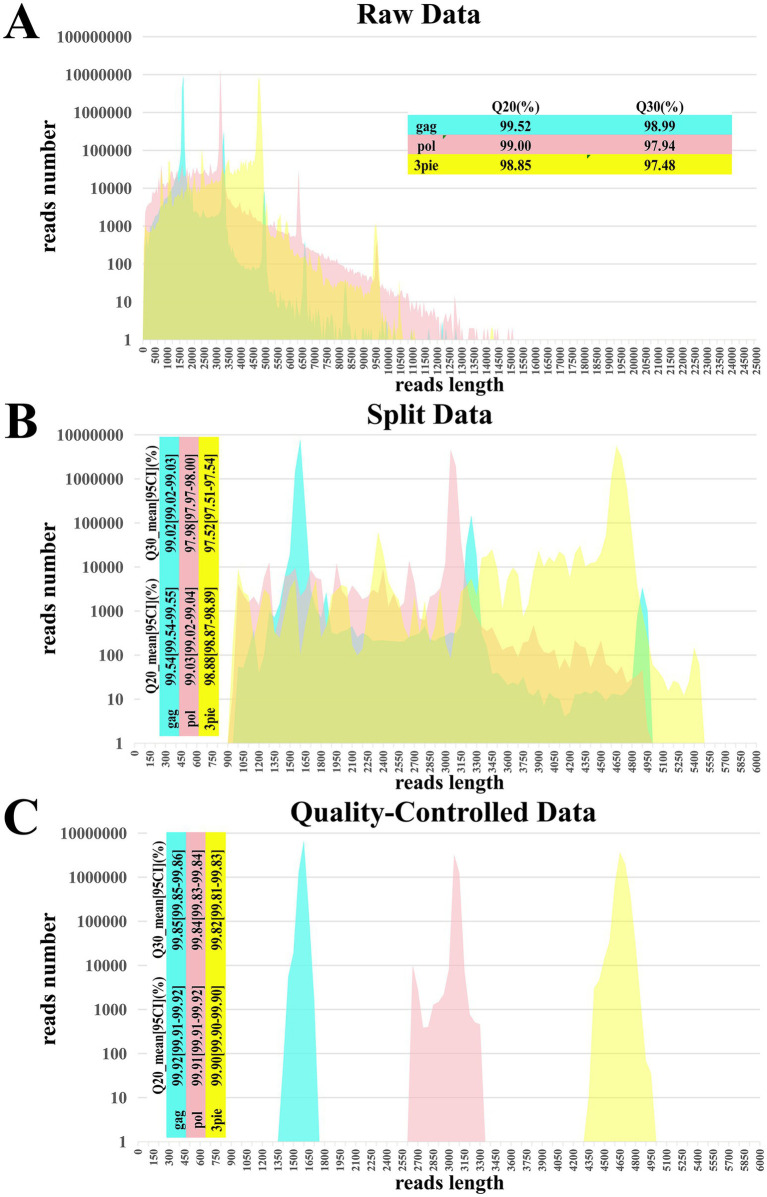
Comparative analysis of PacBio sequencing data quality across processing stages. This figure presents a comparative analysis of sequencing data quality across three processing stages of a PacBio run, evaluated by total data volume and Q20/Q30 percentages (common read quality metrics). **(A)** Raw data; **(B)** split data; **(C)** quality-controlled data.

As shown in Figure 2A, merging the sequencing results from multiple SMRT cells for each genomic fragment generated a total of ‌14,611,575‌, ‌16,833,878‌, and ‌19,046,293‌ CCS reads (raw data) for the *gag*, *pol*, and 3pie fragments, respectively (*N* = 728 + 8). The average sequencing quality scores (Q30%) for these fragments were ‌98.99% (*gag*)‌, ‌97.94% ‌(*pol*), and ‌97.48% (3pie)‌, respectively. The data were primarily distributed around the full-length amplicon size range, with secondary peaks observed at integer multiples of the primary peak due to incomplete separation of polymerase-read sequences. Additionally, some amplicon fragments were present in the data.

Following sample demultiplexing with the *lima* software, the mean Q30 scores with 95% confidence intervals (Q30_mean_[95%CI], %) ‌remained largely stable. To maximize the positive predictive value (PPV) of *lima* for sample demultiplexing, the three fragments retained 66.19% (9,669,443/14,611,575), 40.00% (6,733,052/16,833,878), and 56.93% (10,844,988/19,046,293) of CCS reads (split data), respectively. Subsequent validation confirmed zero misassignment within these retained datasets. At this point, a small portion of the data has not been demultiplexed due to the lack of complete barcodes and amplification primers (Figure 2B).

However, after primary data filtering through the bioinformatics pipeline, the Q30_mean_[95%CI] ‌improved significantly. Specifically, for the 3pie fragment, Q30_mean_[95%CI] increased from ‌97.52% [97.51–97.54]‌ pre-filtering to ‌99.82% [99.81–99.83]‌ post-filtering. Similar enhancements were observed in Q30_mean_[95%CI] and Q20_mean_[95%CI] for the other two fragments. At this stage, the retained data comprised 83.47% (8,071,870/9,669,443), 66.30% (4,464,114/6,733,052), and 62.45% (6,773,199/10,844,988) of the respective datasets (quality-controlled data in Figure 2C). All reads with significant differences in length or poor base quality were excluded.

Collectively, these results demonstrate that the ‌filtered CCS reads‌ possess adequate data volume‌ and ‌high sequencing quality‌, thereby meeting the requirements for subsequent HIV ‌quasispecies-level sequence analysis‌.

### Systematic assessment of recombination and variation in amplification-sequencing workflow

**Figure 3:**
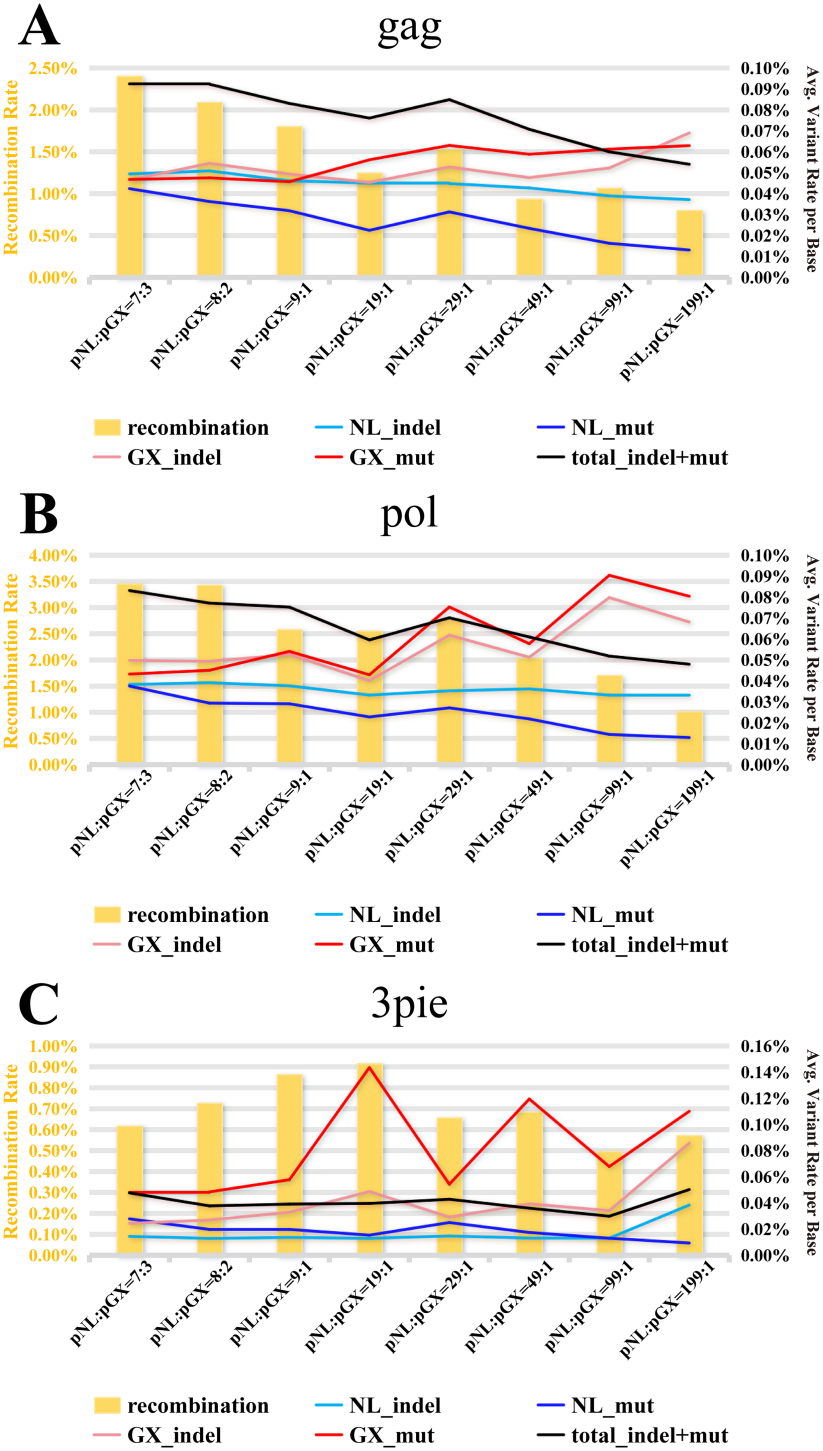
Comprehensive error profiling of amplified sequencing reads across quality control stages. This figure depicts the recombination frequency and nucleotide variation rates (including indels and mutations) observed in amplified PacBio sequencing reads from eight mixed-template samples (pNL:pGX = 7:3; 8:2; 9:1; 19:1; 29:1; 49:1; 99:1; 199:1). **(A–C)** represent the *gag*, *pol*, and *3pie* regions, respectively.

As illustrated in Figure 3, observed recombination rates for *gag* and *pol* fragment reads varied from 0.8 to 3.5%, demonstrating an inverse relationship with increasing template ratios (pNL:pGX = 7:3 to pNL:pGX = 199:1). The 3pie fragment consistently exhibited lower recombination rates (<1%) with no discernible patterns, potentially attributable to the 3pie region being less conserved than *gag* and *pol*—two highly conserved HIV genomic regions where template sequence homology facilitates recombination. Notably, the vast majority of recombinant reads had a uniqueness value of 1 (i.e., each recombinant read was detected only once), making them prone to exclusion during subsequent bioinformatic filtering. Following exclusion of all recombinant reads, indels and mutations in the remaining reads were quantified (Figure 3). Results demonstrated overall variant frequencies below 0.1% in the *gag* and *pol* regions. As template ratio divergence increased, low-abundance templates exhibited a slight upward trend in variant frequency, while high-abundance templates showed a slight downward trend. The 3pie fragments maintained variant frequencies below 0.05% without distinct patterns. Additionally, variant frequencies were generally marginally higher in low-abundance versus high-abundance templates. This bias may stem from PCR artifacts, including stochastic drift, bottleneck effects, and molecular randomness, where bottleneck-amplified early replication errors elevate variants in scarce templates, while molecular redundancy suppresses them in dominant templates. The test results demonstrated that the amplification and sequencing workflow exhibited minimal read-level recombination or variation attributable to systematic errors.

### Sensitivity analysis of clustering parameters‌

**Figure 4:**
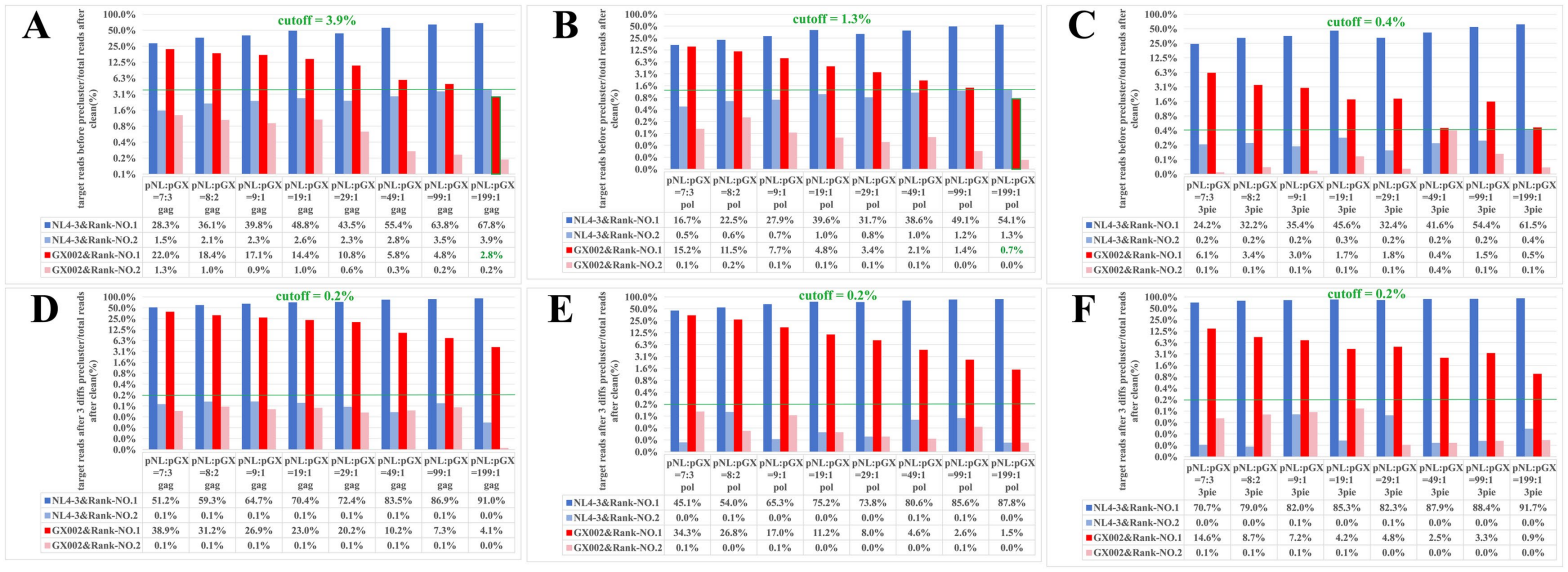
Sensitivity analysis of clustering parameters in HIV-1 quasispecies. This figure presents the proportion of reads from the top 2 ranked clusters for eight different standard amplification templates mixed at various ratios (pNL:pGX = 7:3; 8:2; 9:1; 19:1; 29:1; 49:1; 99:1; 199:1). **(A–C)** Data before preclustering. **(D–F)** Data after preclustering with 3diff parameters.

We conducted a sensitivity analysis on the parameters of the mothur.precluster module and the cutoff value for final quasispecies read screening (Schloss, 2020). Based on the read variation rate results from standard samples, it was further inferred that the average number of variations (diffs) per read is approximately 1–3. Therefore, after processing the sequencing results of standard samples through read alignment, we used the mothur.precluster module with 1–3 diffs (i.e., nucleotide differences) as the clustering parameters to group the reads. As shown in Figures 4A–C, without mothur.precluster processing, no cutoff value could be determined to effectively distinguish the two correct plasmid reads (pNL4-3 and pGX002) from erroneous ones in 8 groups of standard samples. The proportion of erroneous reads from the high-abundance quasispecies in the standard samples (with pNL:pGX = 49:1, 99:1, 199:1) was higher than or close to that of correct reads from the low-abundance quasispecies. When using 1 or 2 diffs parameters in mothur.precluster, although a cutoff value was identifiable to separate and eliminate erroneous reads, small numbers of erroneous sequences and low-abundance correct plasmid sequences remained in similar proportions – making erroneous sequence screening challenging. Under these parameter settings (1 or 2 diffs), the cutoff value ranged unstably between 0.4 and 3.9% (Supplementary Figure S3). However, with the 3 diffs parameter in mothur.precluster, all erroneous reads were effectively filtered at a 0.2% cutoff value (the 99.99% upper reference limit for the maximum error read proportion across all groups was 0.1888%), retaining only the correct plasmid reads (Figures 4D–F).

Representative data from the 3pie fragment (pNL:pGX = 7:3, Figure 4F) processed with 3-diffs mothur.precluster are shown. Alignment of the top 20 reads against reference plasmid sequences (Supplementary Figure S4) demonstrates complete discrimination between mutated and authentic reads at the 0.2% cutoff threshold.

In summary, the optimal parameters and cutoff threshold identified were 3 diffs and 0.2%, respectively.

### Analysis of consistency between approximate quasispecies abundance calculations and template abundance

For 8 sets of standard samples, after processing the reads with precluster at a 3diffs threshold followed by final filtering using a 0.2% cutoff value, each standard sample yielded 2 quasispecies consistent with the template plasmids. We calculated the approximate abundance of these two quasispecies and conducted a consistency analysis between the calculated abundances and the original template plasmid ratios. As shown in Supplementary Figure S5, the overall trend consistency between the two was high (*pol* > 3pie > *gag*). The abundance data consistency of the standard samples for the 3pie fragment decreased significantly when the two template ratios were close. The abundance data consistency of the low-abundance templates in the standard samples for the *gag* fragment showed obvious fluctuations. The primary reason may be the variation in amplification efficiency between different templates, leading to changes in the abundance of the corresponding amplified products. However, overall, the calculated quasispecies abundances of the 3 fragments were approximately consistent with the original template ratios.

### PacBio HiFi and Sanger NFLG sequences show phylogenetic congruence in HIV-1 quasispecies

To evaluate the concordance of NFLG quasispecies sequences obtained via PacBio sequencing versus conventional Sanger methodology, we selected a representative set of 10 samples for which NFLG quasispecies data had previously been generated via single-genome amplification (SGA) followed by Sanger sequencing (Salazar-Gonzalez et al., 2008). We aligned HIV-1 NFLG quasispecies sequences from both platforms with reference sequences to infer maximum-likelihood (ML) phylogenetic trees.

**Figure 5:**
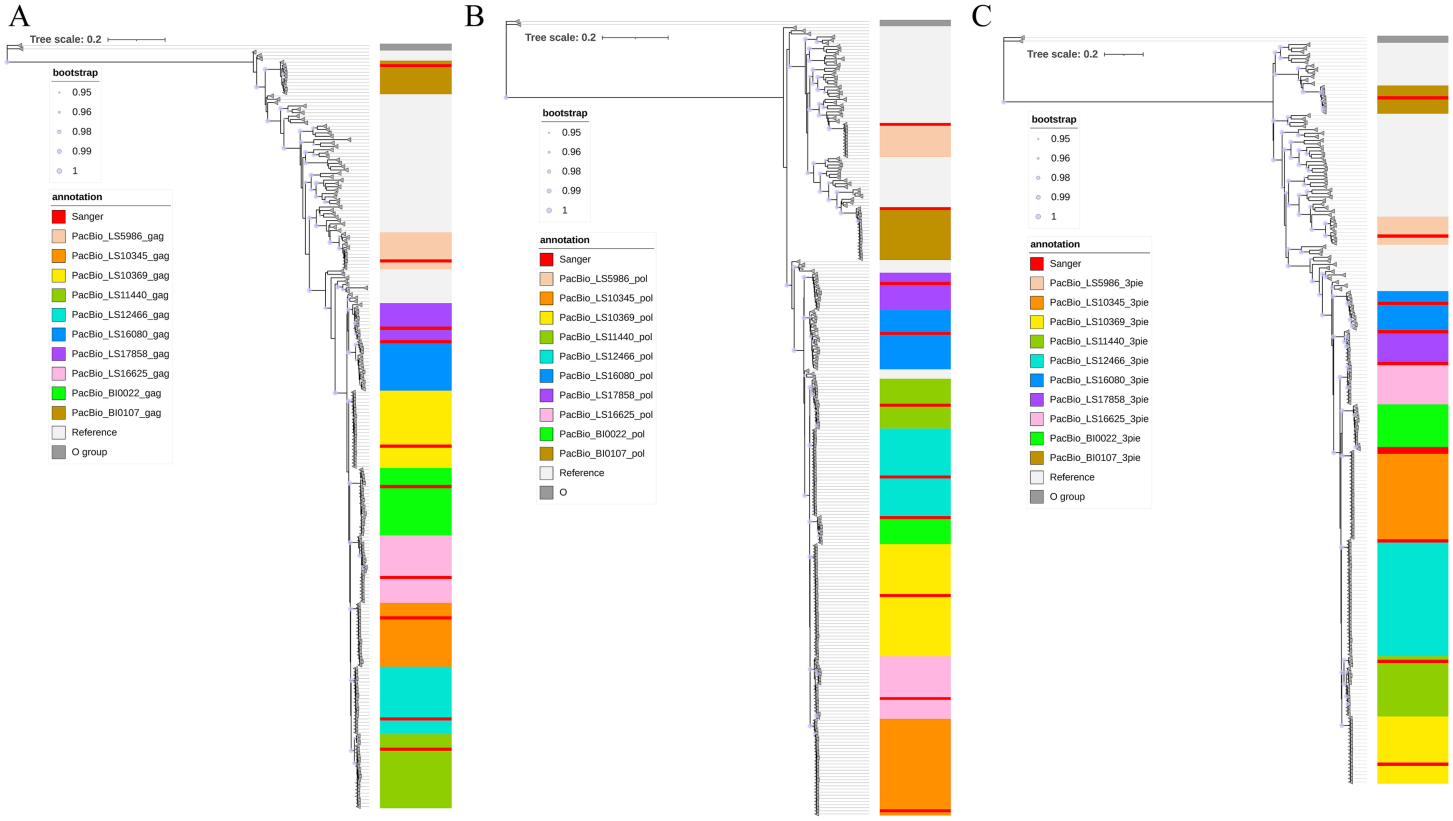
Phylogenetic consistency of PacBio vs. SGA Sanger NFLG sequences. The ML tree construction results of *gag*, *pol* and 3pie fragments are shown in **(A–C)** respectively; different samples are distinguished by color backgrounds, with the red background is the quasispecies sequence obtained by single-genome amplification Sanger sequencing of each sample.

As shown in Figure 5, paired sequencing results from each sample consistently formed monophyletic clusters within the *gag*, *pol*, and 3pie regions. No significant differences were detected between the two sequencing platforms.

In a small subset of samples, Sanger-derived sequences occupied basal positions within their respective monophyletic clades. This likely arose from artificial quasispecies sequence chimerism introduced during secondary SGA re-amplification and subsequent *de novo* contig assembly—a procedure required when ambiguous electrophoretic traces (e.g., double peaks) are encountered in primary Sanger sequencing reactions. Such recombinant artifacts or shared ancestral polymorphic motifs may be phylogenetically interpreted as sequences closer to the inferred root of all quasispecies. Importantly, PacBio-derived quasispecies reads, when processed through our bioinformatics pipeline, yielded single, precise, full-length amplicon sequences—thus avoiding the aforementioned technical artifacts. This highlights the superior fidelity and methodological robustness of the long-read approach.

### PacBio HiFi vs. Sanger SGA NFLG sequencing in HIV-1 dual infection: consistency and superiority in quasispecies analysis

Among the included samples, one previously confirmed as dual infection through SGA and Sanger sequencing was re-examined. Subtyping and recombination analysis of quasispecies from both methodologies demonstrated complete concordance for CRF80_0107 in all *gag* and *pol* fragments. However, divergent results emerged in the 3pie fragment (Supplementary Figure S6).

The conventional SGA-followed Sanger sequencing approach identified 5 quasispecies: one CRF80_0107, and four URF_0180 (URF_0107) exhibiting identical recombination patterns. PacBio sequencing identified 18 distinct quasispecies with quantitative abundance data: CRF80_0107 (16.2%), URF_0180 (71.8%; displaying recombination patterns consistent with Sanger results), and a novel recombination pattern (NO.5, NO.16, NO.17; collectively 11.9%). Notably, among the 13 PacBio-derived quasispecies (71.8% cumulative abundance) that matched Sanger-identified patterns, two quasispecies (NO.4 [10.8%] and NO.6 [9.1%]) revealed subtle but significant recombination breakpoint polymorphisms. This molecular evidence indicates at least two independent recombination events between CRF80_0107 and heterologous subtypes, with potential ongoing recombination dynamics that were undetectable by conventional methods.

The comprehensive profiling achieved through PacBio sequencing demonstrated unequivocal superiority over the Sanger-SGA methodology in both the dual infection identification and the recombination patterns resolution, providing enhanced sensitivity (18 vs. 5 quasispecies detected) and quantitative capability (abundance measurements) while uncovering previously undetected recombination complexity.

### Efficacy analysis of the method for extracting viral quasispecies sequences using the bioinformatics pipeline

**Figure 6:**
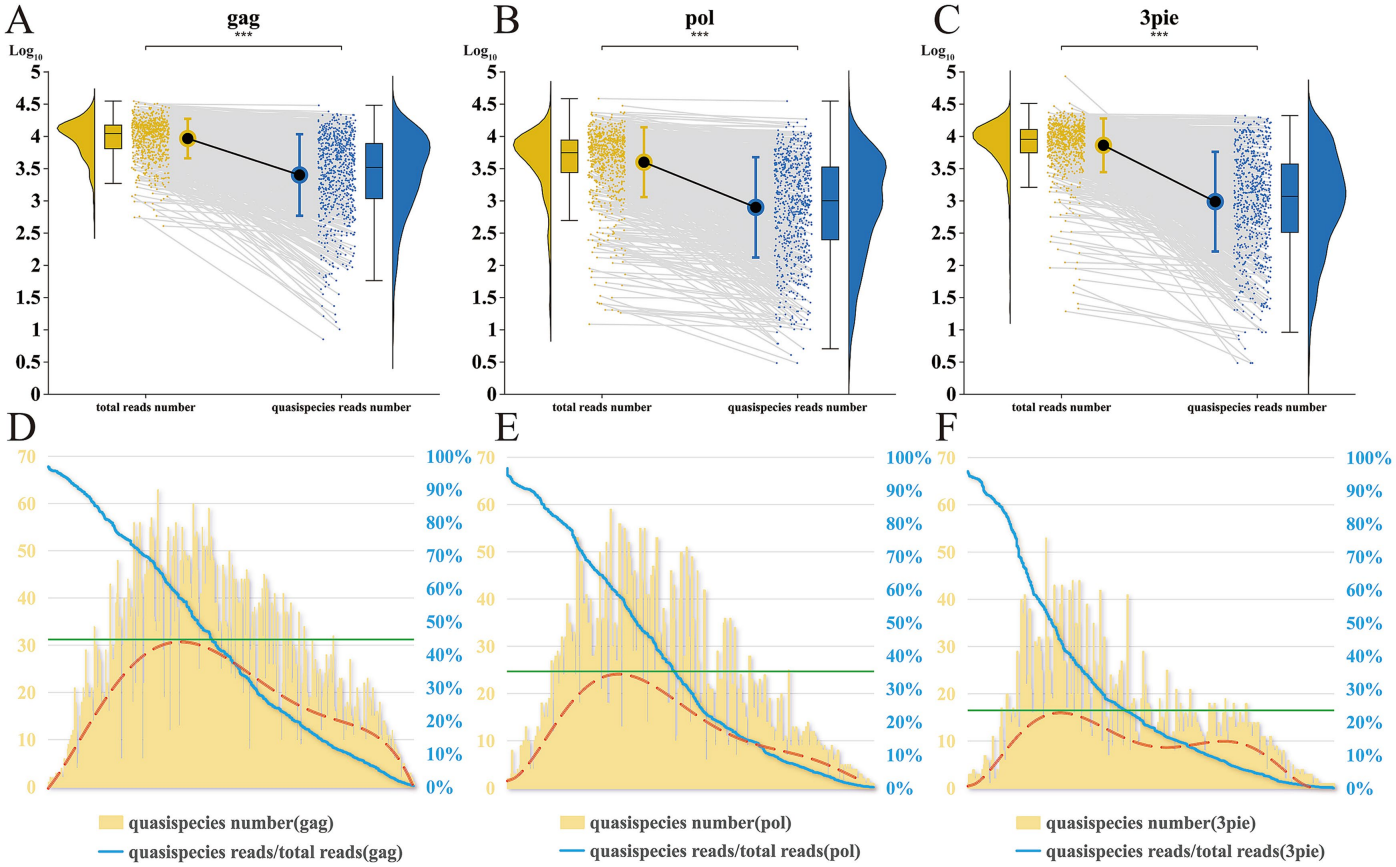
Efficacy analysis of viral quasispecies extraction bioinformatics pipeline. **(A–C)** Comparison of total reads versus quasispecies reads: Boxplots show data distribution; dots represent individual samples; ***p < 0.001 (Wilcoxon test). **(D–F)** Distribution of quasispecies numbers in *gag*, *pol*, and 3pie regions: Yellow bars: Number of clusters; Blue line: Percentage of quasispecies reads/total reads; X-axis: Samples sorted by descending percentage. The red dashed line denotes the trend line for quasispecies numbers, while the green line indicates the maximum extraction efficiency for quasispecies recovery.

Following filtering CCS reads, the mean log reduction in retained quasispecies reads for *gag*, *pol*, and 3pie genomic fragments was 0.5664 (95% CI [0.5284, 0.6044]), 0.7041 (95% CI [0.6898, 0.7184]), and 0.8770 (95% CI [0.8270, 0.9270]), respectively (Figures 6A–C). Although a small subset of samples retained only a limited number of quasispecies reads, all samples contained at least one quasispecies. Ultimately, we obtained 13,537 (*gag*), 9,071 (*pol*), and 6,386 (3pie) quasispecies sequences, along with their approximate abundance data.

To further evaluate the pipeline’s efficacy, we analyzed the relationship between the retention ratio (proportion of reads retained after mothur.precluster filtering) and the final quasispecies count per sample, with samples sorted by descending retention ratio (Figures 6D–F). When the retention ratio exceeded approximately 60%, the quasispecies count was inversely correlated with the retention ratio. Conversely, as the retention ratio dropped below 50%, the quasispecies count decreased proportionally. This pattern emerges because PCR amplification generates fewer erroneous amplicons (from mutations and recombination) when the template is relatively homogeneous, whereas increasing HIV-1 quasispecies template complexity elevates error rates and reduces retention. Consequently, when the actual quasispecies count surpassed a threshold, the retention ratio declined sharply, with most subdominant quasispecies reads falling below the predefined 0.2% cutoff and thus failing to pass filtering. The Bioinformatics Pipeline achieved peak efficacy at a retention ratio of approximately 50–60%, enabling the recovery of mean 31, 25, 16 quasispecies of *gag*, *pol* and 3pie, respectively.

Our analyses revealed distinct loss patterns of subdominant quasispecies under PCR bottlenecking, highlighting the bioinformatics pipeline’s ability to mitigate diversity erosion during HIV-1 genomic amplification. Crucially, samples that retained fewer than five quasispecies constituted only a small minority, underscoring the method’s robustness in preserving viral heterogeneity.

### Subtyping discordance and dual infection detection in NFLG-based HIV-1 analysis

**Figure 7:**
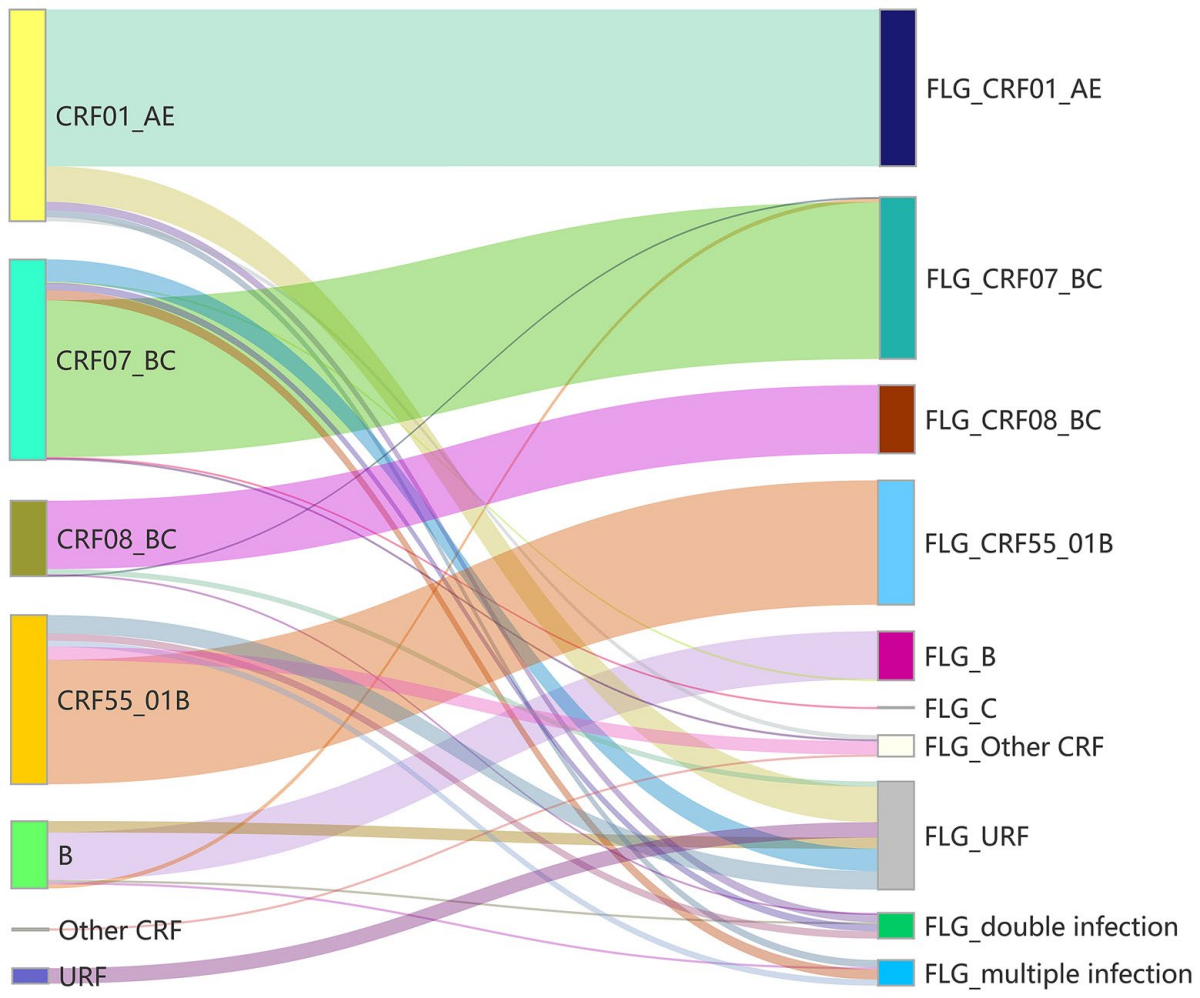
Subtype correction by NFLG quasispecies sequences (PacBio). Left panel: Original HIV-1 subtype classification based on Sanger sequencing of the *pol* fragment (HXB2 coordinates: 2253–3,440). Right panel: Corrected subtype assignments derived from PacBio-generated near full-length genomic (NFLG) quasispecies sequences using our optimized pipeline. The term “multiple infection” in this figure refers to the detection of three or more subtypes or novel recombinant types.

Comprehensive subtyping and recombination analyses were performed on NFLG quasispecies across 728 HIV-1 samples. Results showed discordant subtyping outcomes in 23.5% (171/728) of samples compared with previous classifications from drug resistance surveillance (Figure 7; Supplementary Table S3). Among the five predominant HIV-1 strains in China, discordance rates were as follows: subtype B: 28.8% (19/66), CRF01_AE: 26.0% (54/208), CRF07_BC: 21.8% (43/197), CRF08_BC: 9.5% (7/74), and CRF55_01B: 26.5% (44/166). Furthermore, dual infections were identified in 29.2% (50/171) of discordant samples. These findings highlight the improved ability of the NFLG quasispecies bioinformatics pipeline to accurately perform HIV-1 subtyping and genetic diversity profiling, particularly in detecting complex dual infections.

## Discussion

HIV-1 exists as highly abundant quasispecies, forming an extraordinarily complex template due to its high mutation rate and prolonged latency (Domingo et al., 2012; Cuevas et al., 2015). Traditional sequencing relies on SGA for template dilution, followed by Sanger sequencing of amplicons—a process that drastically increases workload and hinders large-scale NFLG sequencing of HIV-1 (Salazar-Gonzalez et al., 2008; Butler et al., 2009). Illumina short-read sequencing platforms (e.g., MiSeq, NextSeq) offer wide accessibility and high throughput, leading to their widespread use in virology laboratories for HIV-1 NFLG research (Gall et al., 2012; Ode et al., 2015). While Illumina-based NFLG methodologies enable high-throughput detection of low-frequency variants without relying on SGA, they nevertheless possess several inherent limitations (Supplementary Table S4). Critically, these methods fail to accurately resolve the complete linked sequences of individual quasispecies or amplicons. NFLG data are typically generated as consensus sequences, and reconstructing the ~9-kb NFLG from short reads or contigs tends to introduce biases—particularly in hypervariable regions such as *env* and at recombination breakpoints regions—while complicating the differentiation between true viral genomic recombination and PCR-mediated recombination. Additionally, accurate detection of dual infections also poses a challenge. These approaches often require complex bioinformatics pipelines, which lead to inconsistencies in results among various studies. Furthermore, Illumina platforms offer only a marginal cost advantage over Sanger sequencing. Recent advances in long-read sequencing have improved platforms for HIV-1 genotyping and quasispecies research. For instance, Wright et al. (2021) described a classical nanopore sequencing approach, yet it still requires limiting dilution. Mori et al. (2022) further developed an analytical pipeline for nanopore-derived HIV-1 sequences, inferring NFLG consensus sequences with error rates of 0.011–0.056%. However, significant limitations persist. Minority reads from nanopore sequencing are often masked by dominant sequences during consensus building, potentially failing to capture the original viral quasispecies diversity, particularly for minor variants, highlighting its ongoing unsuitability for accurate quasispecies profiling. Additionally, nanopore single-molecule sequencing (with ~92% accuracy) requires extra validation due to homopolymer regions (e.g., polypurine tracts) in the HIV-1 genome to ensure single-base resolution reliability and avoid erroneous indels (Lu et al., 2016; Chen et al., 2021; Ranasinghe et al., 2022; Liu-Wei et al., 2024). In contrast, Gisele Umviligihozo et al. integrated the PacBio Single Molecule Real-Time (SMRT) RSII platform with the MDPseq workflow to define transmitted founder (TF) virus sequences, demonstrating superior capability to identify inter-subtype recombinants in NFLG sequencing.

In this study, we developed and optimized a HiFi amplification method for NFLG of HIV-1 quasispecies. This approach eliminates the need for conventional SGA, significantly enhancing valid amplicon yield. Validation using standardized reference strains and clinical plasma samples consistently demonstrated robust amplification efficiency (>80%) for specimens with viral loads exceeding 1,000 copies/mL. Large-scale amplification trials involving China’s six predominant circulating strains (*N* = 894) confirmed broad applicability across diverse subtypes. Furthermore, we developed a specialized bioinformatics pipeline for processing PacBio-derived HIV-1 quasispecies sequencing data. The workflow features exceptional simplicity and reproducibility, with executable command-line examples provided in the Supplementary materials.

The efficacy of this bioinformatics pipeline in quasispecies sequence extraction derives from its innovative dual-phase clustering and filtering approach. Within HiFi amplification sequencing systems, error-free reads generated through flawless template amplification constitute the majority of post-filtering datasets (Wang et al., 2025). However, systematic biases often induce significant variability in abundance distribution among different quasispecies reads, complicating both quasispecies isolation and quantitative analysis (Jansz and Faulkner, 2024). The clustering phase employs Mothur’s pre.cluster module to strategically group reads containing minor errors (1–3 nucleotide discrepancies) with their corresponding dominant error-free quasispecies reads. This process systematically processes filtered unique reads in descending abundance order: each distinct read undergoes iterative matching within predefined diffs threshold, followed by merging with the highest-abundance qualifying reads. Figure 1B shows the schematic diagram of the principle of this method. Although this results in the loss of a minority subset of quasispecies, the impact on subsequent sequence analyses is insignificant. Notably, the merging process can potentially amplify the relative abundance of certain error-containing reads through incorporation of low-frequency reads (Huse et al., 2010). To mitigate this, we implement a rigorously validated cutoff threshold to discriminate genuine quasispecies reads from error reads. The final dataset—comprising quasispecies reads meeting this stringent criterion—demonstrates the protocol’s superior resolution in quasispecies-level characterization. A key advantage is its preservation of raw sequencing reads in their native state—free from artificial manipulations such as consensus calling via clustering. Leveraging the intrinsic accuracy of the HiFi system (Wang et al., 2025), where error-free amplicon reads consistently outnumber erroneous ones, our pipeline employs systematic filtering and merging procedures to retain most authentic quasispecies sequences while simultaneously capturing their approximate abundance profiles. These comprehensive datasets, encompassing both sequence variants and their relative abundances, enhance the ability‌ to resolve evolutionary trajectories within HIV-1 genomes. Performance was further amplified by integrating the state-of-the-art PacBio Revio sequencing platform, which offers order-of-magnitude improvements in throughput, computational efficiency, and cost-effectiveness compared to earlier systems. Its expanded capacity of 25 million zero-mode waveguides (ZMWs) proved particularly critical for large-scale acquisition of NFLG quasispecies sequences. This technological leap directly enabled our pipeline’s exceptional sensitivity—successfully identifying 28,994 distinct quasispecies from an initial pool of 50,491,746 circular consensus sequencing (CCS) reads.

Our in-depth analysis of the quasispecies sequences yielded two significant findings: first, 23.5% (95% CI: 21.8–25.3%) of initial HIV-1 subtype assignment from conventional methods were incorrect; second, 29.2% (95% CI: 26.1–32.6%) of the corrected cases were confirmed as inter-subtype dual infections. Statistical validation confirms that our approach outperforms conventional methods in HIV-1 subtyping (*p* < 0.01, χ^2^ test). Beyond accuracy, these findings reveal a previously underestimated prevalence of novel recombinant strains and cryptic dual infections circulating in the study population—phenomena likely masked by older methodologies (Wan et al., 2025). These high detection rates of complex infection patterns underscore the two critical implications: the clinical utility of our high-resolution sequencing strategy, and the pressing need to strengthen surveillance of HIV-1 genetic diversity in molecular epidemiology programs.

This study has several limitations that merit discussion. (a) Sample size and multiplexing constraints in PacBio sequencing‌. The PacBio-based approach for acquiring HIV-1 NFLG quasispecies is suboptimal for small-scale studies due to its high per-sample resource costs. While multiplexing >1,000 samples per SMRT Cell is feasible, it complicates demultiplexing due to barcode limitations. Additionally, large multiplexed pools risk representation bias: imbalanced amplicon pooling can lead to uneven sequencing coverage across samples. We recommend pooling 300–1,000 samples per run as an optimal compromise. (b) Suboptimal NFLG amplicon design‌. Our NFLG amplicon strategy remains imperfect: PacBio sequencing shows low ZMW loading efficiency (∼20–30%) for shorter *gag*/*pol* fragments, requiring more SMRT cells. We attempted direct HiFi-PCR amplification of the 5′-half genome fragment (HXB2: 454–5,265, 4,812 bp) as an alternative approach, but this produced suboptimal results.‌ However, the aforementioned limitations have now been effectively addressed. PacBio Revio platform’s SPRQ sequencing reagents (upgraded from SPRA reagents), launched in Q1 2025, significantly optimized sequencing performance for small fragments. Our unpublished sequencing data demonstrated that both *gag* and *pol* fragments achieved 10 million reads per cell. (c) Dependency on HiFi-PCR fidelity‌. Quasispecies recovery efficacy relies critically on HiFi-PCR accuracy: enzymes with ≥20-fold higher fidelity than Taq polymerase are required for reliable results. While ultra-high-fidelity polymerases improve pipeline performance, they substantially increase costs. For complex quasispecies samples, template switching during PCR is unavoidable. Moreover, HiFi polymerases have a narrower dynamic range than conventional Taq polymerase, leading to slightly lower PCR positivity rates and occasional data loss in some specimens. We anticipate that ongoing advancements in PacBio sequencing technology and next-generation HiFi polymerases will refine HIV-1 NFLG quasispecies acquiring protocols, thereby advancing viral genomics research at the quasispecies level.

Collectively, our novel methodology enables efficient, high-precision identification of HIV-1 NFLG quasispecies from plasma-derived RNA. Notably, this approach offers distinct advantages in resolving complex viral population dynamics, ‌particularly‌ in characterizing quasispecies within clinically challenging dual-infection specimens, where traditional methods often fall short. The streamlined workflow, deployed on PacBio’s high-throughput Revio platform, supports robust detection across large sample sets, addressing scalability barriers in prior quasispecies research. By generating both high-fidelity sequences and approximate abundance profiles, our pipeline significantly enhances resolution for evolutionary reconstruction. Moreover, its practical utility is reinforced by command-line executable protocols, ensuring accessibility for broader adoption. These advances collectively strengthen molecular epidemiological surveillance: by empowering efforts to track HIV-1 transmission networks, elucidate evolutionary pathways, and monitor the emergence of antiretroviral resistance mechanisms. In doing so, the method bridges technical gaps in high-resolution viral genomics, facilitating deeper insights into HIV-1’s adaptive dynamics.

## Data Availability

The datasets presented in this study can be found in online repositories. The names of the repository/repositories and accession number(s) can be found at: https://www.ncbi.nlm.nih.gov/genbank/, OQ513542, OQ513543, OQ513556, OQ513565, OQ513514, OQ513521, OQ513498; https://nmdc.cn, SUB1761744349403.

## Footnotes

1
https://comet.lih.lu/

2
http://jphmm.gobics.de/submission_hiv

3
https://www.hiv.lanl.gov/components/sequence/HIV/crfdb/crfs.comp
