# Supplementary material for: PacBio high-fidelity near full-length genome sequencing for HIV-1 quasispecies: methodological framework and validation
Source: Front Microbiol. 2026 Jan 13;16:1738027. doi: 10.3389/fmicb.2025.1738027 (PMC12835203; doi:10.3389/fmicb.2025.1738027)
Supplement: Supplementary file 1 [file Data_Sheet_1.zip › markdown-file.html]

Command guidance


# Command guidance

**All command-line operations use
`%sample.name%_gag.fastq`
`%sample.name%_pol.fastq`
`%sample.name%_3pie.fastq` as the designated sample
name.**

## 1. CCS Read Directional Processing

**Directional standardization of CCS-reads was implemented
using custom Python scripts, converting all demultiplexed samples to
uniform 5’→3’ orientation.**

## 2. CCS-Reads Data Filtering

**2.1 Base Quality Filtering**  
**This procedure was implemented using
`fastp version 0.20.1` with the following command-line
parameters:**

- gag region processing  
  `fastp -i %sample.name%_gag.fastq -o %sample.name%_gag.fastq.q40u1.fq -h %sample.name%_gag.fastq.q40u1.html -q 40 -u 1`
- pol region processing  
  `fastp -i %sample.name%_pol.fastq -o %sample.name%_pol.fastq.q40u1.fq -h %sample.name%_pol.fastq.q40u1.html -q 40 -u 1`
- 3pie region processing  
  `fastp -i %sample.name%_3pie.fastq -o %sample.name%_3pie.fastq.q40u1.fq -h %sample.name%_3pie.fastq.q40u1.html -q 40 -u 1`

`-q 40`: Sets Phred quality threshold (Q40) for base
trimming.  
`-u 1`: Filters reads with >1% low-quality bases.  
Dual-output reports: JSON/HTML formats for quality metrics
visualization.

**2.2 File Format Conversion: FASTQ to FASTA**  
**This procedure was executed using
`seqkit Version: 2.1.0` with the following command
syntax:**

- gag region processing  
  `seqkit fq2fa %sample.name%_gag.fastq.q40u1.fq > %sample.name%_gag.fastq.q40u1.fq.fasta`
- pol region processing  
  `seqkit fq2fa %sample.name%_pol.fastq.q40u1.fq > %sample.name%_pol.fastq.q40u1.fq.fasta`
- 3pie region processing  
  `seqkit fq2fa %sample.name%_3pie.fastq.q40u1.fq > %sample.name%_3pie.fastq.q40u1.fq.fasta`

**2.3 Barcode and Primer Removal**  
**This procedure was performed using
`cutadapt version 3.5` with region-specific adapter trimming
configurations:**

- gag region processing  
  `cutadapt -g TGACTAGCGGAGGCTAGAAGG -o %sample.name%_gag.cutadapt_g.fasta %sample.name%_gag.fastq.q40u1.fq.fasta -e 2 -n 1 -j 16`  
  `cutadapt -a GGAAACCAAAAATGATAGGGGGAA -o %sample.name%_gag.fastq.q40u1.fq.fasta.cutadapt.fasta %sample.name%_gag.cutadapt_g.fasta -e 2 -n 1 -j 16`
- pol region processing  
  `cutadapt -g ACTGARAGACAGGCTAATTTTTTAGGGA -o %sample.name%_pol.cutadapt_g.fasta %sample.name%_pol.fastq.q40u1.fq.fasta -e 2 -n 1 -j 16`  
  `cutadapt -a TAAGYTCAGAAGTACAYATCCCACTAGGAG -o %sample.name%_pol.fastq.q40u1.fq.fasta.cutadapt.fasta %sample.name%_pol.cutadapt_g.fasta -e 2 -n 1 -j 16`
- 3pie region processing  
  `cutadapt -g GGGTTTATTACAGGGACAGCAGAG -o %sample.name%_3pie.cutadapt_g.fasta %sample.name%_3pie.fastq.q40u1.fq.fasta -e 2 -n 1 -j 16`  
  `cutadapt -a GGAACCCACTGCTTAAGCCTCA -o %sample.name%_3pie.fastq.q40u1.fq.fasta.cutadapt.fasta %sample.name%_3pie.cutadapt_g.fasta -e 2 -n 1 -j 16`

Region-specific primers target conserved HIV-1 sequences (gag, pol,
3pie) with degenerate bases (R= A/G, Y= C/T) accommodating viral
diversity.  
`-e 2`: absolute number of errors for full-length adapter
match (if number is an integer >= 1) (optimized for viral sequence
heterogeneity).  
`-n 1`: Processes single adapter pairs per execution
(efficiency-focused).  
`-j 16`: Utilize 16 CPU threads for parallel processing.

**2.4 Sequence Length Filtering**  
**This procedure was implemented using
`seqkit Version: 2.1.0` with region-specific length
thresholds:**

- gag region length selection (1450-1750 bp)  
  `seqkit seq -m 1450 -M 1750 %sample.name%_gag.fastq.q40u1.fq.fasta.cutadapt.fasta > %sample.name%_gag.fastq.q40u1.fq.fasta.cutadapt.fasta.lengthcut.fasta`
- pol region length selection (2650-3350 bp)  
  `seqkit seq -m 2650 -M 3350 %sample.name%_pol.fastq.q40u1.fq.fasta.cutadapt.fasta > %sample.name%_pol.fastq.q40u1.fq.fasta.cutadapt.fasta.lengthcut.fasta`
- 3pie region length selection (4400-4900 bp)  
  `seqkit seq -m 4400 -M 4900 %sample.name%_3pie.fastq.q40u1.fq.fasta.cutadapt.fasta > %sample.name%_3pie.fastq.q40u1.fq.fasta.cutadapt.fasta.lengthcut.fasta`

`-m`/`-M` flags define minimum/maximum length
cutoffs optimized for HIV-1 genomic regions.

**2.5 Sequence Header Modification**  
**This procedure was performed using GNU `sed` to
append sample identifiers to CCS-read headers:**

- gag region processing  
  `sed -i 's/ccs/ccs%sample.name%/g' %sample.name%_gag.fastq.q40u1.fq.fasta.cutadapt.fasta.lengthcut.fasta`
- pol region processing  
  `sed -i 's/ccs/ccs%sample.name%/g' %sample.name%_pol.fastq.q40u1.fq.fasta.cutadapt.fasta.lengthcut.fasta`
- 3pie region processing  
  `sed -i 's/ccs/ccs%sample.name%/g' %sample.name%_3pie.fastq.q40u1.fq.fasta.cutadapt.fasta.lengthcut.fasta`

Appends unique sample identifiers ‘%sample.name%’ to CCS-read headers
while preserving original ‘ccs’ tags.

**2.6 Post-Filtering Read Statistics**  
**This quality control step was performed using
`seqkit Version: 2.1.0` to quantify retained sequencing
reads:**

- `seqkit stats *.lengthcut.fasta > total_reads_count.xlsx -T`

## 3. CCS-Reads Alignment

**3.1 Unique Processing of Identical CCS-Reads**  
**This step utilizes the `unique.seqs` module within
`mothur v.1.47.0` to handle duplicate
sequences.**  
For more information, visit: https://mothur.org/  
**Command-Line Implementation:**

- Initiate the mothur environment. Start the `mothur`
  software by typing:  
  `mothur`

**Within the mothur interface, execute the following commands
for each region-specific dataset:**

- For the gag region:  
  `unique.seqs(fasta=%sample.name%_gag.fastq.q40u1.fq.fasta.cutadapt.fasta.lengthcut.fasta)`
- For the pol region:  
  `unique.seqs(fasta=%sample.name%_pol.fastq.q40u1.fq.fasta.cutadapt.fasta.lengthcut.fasta)`
- For the 3pie region:  
  `unique.seqs(fasta=%sample.name%_3pie.fastq.q40u1.fq.fasta.cutadapt.fasta.lengthcut.fasta)`

The `unique.seqs` command identifies and retains a single
copy of each unique sequence, eliminating exact duplicates. This step
reduces computational load and memory usage for subsequent alignment
analysis.

**3.2 Sequence Alignment**  
**This step was carried out using `MAFFT v7.490` for
aligning the sequences. Command-line examples are provided below:**

- Aligning the gag region sequences  
  `mafft --thread 16 --threadtb 10 --threadit 0 --inputorder --adjustdirection --anysymbol --auto %sample.name%_gag.fastq.q40u1.fq.fasta.cutadapt.fasta.lengthcut.unique.fasta > %sample.name%_gag.fastq.q40u1.fq.fasta.cutadapt.fasta.lengthcut.unique.fasta.mafft.fasta`
- Aligning the pol region sequences  
  `mafft --thread 16 --threadtb 10 --threadit 0 --inputorder --adjustdirection --anysymbol --auto %sample.name%_pol.fastq.q40u1.fq.fasta.cutadapt.fasta.lengthcut.unique.fasta > %sample.name%_pol.fastq.q40u1.fq.fasta.cutadapt.fasta.lengthcut.unique.fasta.mafft.fasta`
- Aligning the 3pie region sequences  
  `mafft --thread 16 --threadtb 10 --threadit 0 --inputorder --adjustdirection --anysymbol --auto %sample.name%_3pie.fastq.q40u1.fq.fasta.cutadapt.fasta.lengthcut.unique.fasta > %sample.name%_3pie.fastq.q40u1.fq.fasta.cutadapt.fasta.lengthcut.unique.fasta.mafft.fasta`

`--thread 16`: Specifies the number of threads (CPU cores)
to use for the main alignment process, optimizing computation
time.  
`--threadtb 10`: Sets the number of threads for
tree-building, which may be adjusted based on system resources.  
`--threadit 0`: Disables threading for iterative refinement,
potentially useful when dealing with large datasets or limited
computational resources.  
`--inputorder`: Preserves the input order of sequences in the
output alignment.  
`--adjustdirection`: Adjusts the direction of gaps to
minimize the number of overhangs.  
`--anysymbol`: Allows the inclusion of any character in
sequence names, enhancing compatibility with diverse datasets.  
`--auto`: Automatically selects the most appropriate
alignment strategy based on input data characteristics.

## 4. Sorting of Unique CCS-reads in Descending Order

**4.1 Count Sorting**  
**Objective: Combine count files from all samples into a single
file, sort the combined file based on sample names (after removing
additional characters) primarily and `total` count
secondarily in descending order, and then regenerate individual
`count_table` files for each sample indexed by their
respective names.**  
**Step-by-Step Commands:**

- Combining Count Files:  
  Merge all count files
  `*.fastq.q40u1.fq.fasta.cutadapt.fasta.lengthcut.count_table`
  into a single `all.count_table`.
- Sorting the Combined File:  
  Sort `all.count_table` using the sample name (with other
  characters removed) as the primary key and the `total` count
  as the secondary key, both in descending order.
- Regenerating Individual Count Tables:  
  Extract sorted count data for each sample and create new
  `count_table` files.

**Command-line Examples for Segmenting Content:**

- `cat -n all.count_table|grep "%sample.name%" >>%sample.name%_gag.fastq.q40u1.fq.fasta.cutadapt.fasta.lengthcut.unique.fasta.mafft.sorted.count_table_uncut`
- `cat -n all.count_table|grep "%sample.name%" >>%sample.name%_pol.fastq.q40u1.fq.fasta.cutadapt.fasta.lengthcut.unique.fasta.mafft.sorted.count_table_uncut`
- `cat -n all.count_table|grep "%sample.name%" >>%sample.name%_3pie.fastq.q40u1.fq.fasta.cutadapt.fasta.lengthcut.unique.fasta.mafft.sorted.count_table_uncut`

**Command-line Examples for Removing Unnecessary
Characters:**

- `awk '{$1="";$4=""; print $0}' %sample.name%_gag.fastq.q40u1.fq.fasta.cutadapt.fasta.lengthcut.unique.fasta.mafft.sorted.count_table_uncut > %sample.name%_gag.fastq.q40u1.fq.fasta.cutadapt.fasta.lengthcut.unique.fasta.mafft.sorted.count_table_uninput`
- `awk '{$1="";$4=""; print $0}' %sample.name%_pol.fastq.q40u1.fq.fasta.cutadapt.fasta.lengthcut.unique.fasta.mafft.sorted.count_table_uncut > %sample.name%_pol.fastq.q40u1.fq.fasta.cutadapt.fasta.lengthcut.unique.fasta.mafft.sorted.count_table_uninput`
- `awk '{$1="";$4=""; print $0}' %sample.name%_3pie.fastq.q40u1.fq.fasta.cutadapt.fasta.lengthcut.unique.fasta.mafft.sorted.count_table_uncut > %sample.name%_3pie.fastq.q40u1.fq.fasta.cutadapt.fasta.lengthcut.unique.fasta.mafft.sorted.count_table_uninput`

**Command-line Examples for Inserting Headers:**

- `awk 'BEGIN{print "Representative_Sequence total"} {print $0}' %sample.name%_gag.fastq.q40u1.fq.fasta.cutadapt.fasta.lengthcut.unique.fasta.mafft.sorted.count_table_uninput > %sample.name%_gag.fastq.q40u1.fq.fasta.cutadapt.fasta.lengthcut.unique.fasta.mafft.sorted.count_table`
- `awk 'BEGIN{print "Representative_Sequence total"} {print $0}' %sample.name%_pol.fastq.q40u1.fq.fasta.cutadapt.fasta.lengthcut.unique.fasta.mafft.sorted.count_table_uninput > %sample.name%_pol.fastq.q40u1.fq.fasta.cutadapt.fasta.lengthcut.unique.fasta.mafft.sorted.count_table`
- `awk 'BEGIN{print "Representative_Sequence total"} {print $0}' %sample.name%_3pie.fastq.q40u1.fq.fasta.cutadapt.fasta.lengthcut.unique.fasta.mafft.sorted.count_table_uninput > %sample.name%_3pie.fastq.q40u1.fq.fasta.cutadapt.fasta.lengthcut.unique.fasta.mafft.sorted.count_table`

By following these steps, we ensure that the count data for each
sample is organized and sorted accurately, facilitating further analysis
and interpretation.

**4.2 Sequence Sorting**  
**This step was implemented using the `sort.seqs`
module within `mothur v.1.47.0` to reorder aligned sequences
based on predefined identifiers:**  
**Processing Workflow:**

- ID File Generation: Consolidated sorted sequence identifiers
  extracted from Section 4.1 outputs. (`all.count_table` after
  `Sorting the Combined File` step.)  
  Created master ID file `ID.all.count_table` containing
  ordered identifiers from all samples.
- Sequence Reordering: Initiate the mothur environment. Start the
  `mothur` software by typing:  
  `mothur`
- Execute sorting for each genomic region  
  `sort.seqs(fasta=%sample.name%_gag.fastq.q40u1.fq.fasta.cutadapt.fasta.lengthcut.unique.fasta.mafft.fasta,accnos=ID.all.count_table)`
  `sort.seqs(fasta=%sample.name%_pol.fastq.q40u1.fq.fasta.cutadapt.fasta.lengthcut.unique.fasta.mafft.fasta,accnos=ID.all.count_table)`
  `sort.seqs(fasta=%sample.name%_3pie.fastq.q40u1.fq.fasta.cutadapt.fasta.lengthcut.unique.fasta.mafft.fasta,accnos=ID.all.count_table)`

## 5. Sequence Pre-Clustering

**This step utilizes the `pre.cluster` Module in
`mothur v.1.47.0` to merge sorted count files and sequence
files while reducing errors sequences.**

- Execution in Mothur Environment:  
  `mothur`
- Pre-cluster sequences for each genomic region with 3-score
  tolerance:  
  `pre.cluster(fasta=%sample.name%_gag.fastq.q40u1.fq.fasta.cutadapt.fasta.lengthcut.unique.fasta.mafft.sorted.fasta,count=%sample.name%_gag.fastq.q40u1.fq.fasta.cutadapt.fasta.lengthcut.unique.fasta.mafft.sorted.count_table,diffs=3)`  
  `pre.cluster(fasta=%sample.name%_pol.fastq.q40u1.fq.fasta.cutadapt.fasta.lengthcut.unique.fasta.mafft.sorted.fasta,count=%sample.name%_pol.fastq.q40u1.fq.fasta.cutadapt.fasta.lengthcut.unique.fasta.mafft.sorted.count_table,diffs=3)`  
  `pre.cluster(fasta=%sample.name%_3pie.fastq.q40u1.fq.fasta.cutadapt.fasta.lengthcut.unique.fasta.mafft.sorted.fasta,count=%sample.name%_3pie.fastq.q40u1.fq.fasta.cutadapt.fasta.lengthcut.unique.fasta.mafft.sorted.count_table,diffs=3)`

`diffs=3`: Allows sequences with ≤3 score differences to
be merged, effectively correcting PCR and sequencing errors while
preserving biological variants. This step is critical for distinguishing
true biological variants from technical noise in viral genomic analyses.
For more information, visit: https://mothur.org/wiki/pre.cluster/

## 6. Post-Clustering Analysis of Viral Quasispecies

**Objective: Merge pre-clustered count tables from all samples
and perform hierarchical sorting for quasispecies
characterization.**

**6.1 Consolidated Count File Processing**

- Combining Count Files：  
  Merge all count files
  `*.fastq.q40u1.fq.fasta.cutadapt.fasta.lengthcut.unique.fasta.mafft.sorted.precluster.count_table`
  into a single file `allprecluster.count_table`.
- Sorting the Combined File:  
  Open `allprecluster.count_table` in Excel. Sort
  `allprecluster.count_table` using the sample name (with other
  characters removed) as the primary key and the `total` count
  as the secondary key, both in descending order.
- Read Count Normalization：  
  The stream editor `sed` performs in-place text substitution
  to integrate filtered read counts from `Section 2.6` into
  sorted file`allprecluster.count_table`.  
  `sed -i 's/%sample.name%/filtered_read_count_number/g' allprecluster.count_table`
- Calculation of Sequence Relative Abundance:  
  \[\text{pre.cluster reads abundance (%)} =
  \left( \frac{\text{Number of pre.clustered reads}}{\text{Total number of
  filtered reads}} \right) \times 100\]

**6.2 Quasispecies Identification and Abundance
Quantification**

- **Quasispecies Identification and Threshold
  criterion:**  
  Retain sequences with `pre-cluster reads abundance` > 0.2%
  (cut off value) as quasispecies sequences.
- **Pivot Table Operations in Excel:**   
  Calculate total reads number of qualifying quasispecies per
  sample.
- **Compute individual quasispecies sequences abundance:**

\[\text{Quasispecies abundance (%)} =
\left( \frac{\text{Number of quasispecies reads}}{\text{Total number of
all quasispecies reads}} \right) \times 100\]

## 7. Implementation Note

**Recommended Workflow:**

- **Per-Sample Command Generation: Utilize Excel templates for
  batch automated generation of all sample-specific command-line
  operations syntax.**
- **Recommended Execution Environment: Perform all computational
  operations on `Ubuntu 22.04.5 LTS`.**
